# Improving the electron spin properties of nitrogen-vacancy centres in nanodiamonds by near-field etching

**DOI:** 10.1038/s41598-018-34158-4

**Published:** 2018-10-26

**Authors:** F. Brandenburg, R. Nagumo, K. Saichi, K. Tahara, T. Iwasaki, M. Hatano, F. Jelezko, R. Igarashi, T. Yatsui

**Affiliations:** 1 School of Engineering, University of Tokyo, Tokyo, Japan; 2 Tokyo Institute of Technology, Tokyo, Japan; 3 Institute of Quantum Optics, Ulm University, Ulm, Germany; 4 QST Future Laboratory, National Institutes for Quantum and Radiological Science and Technology (QST), Chiba, Japan; 5 National Institute of Radiological Sciences (NIRS), National Institutes for Quantum and Radiological Science and Technology (QST), Chiba, Japan

## Abstract

The nitrogen-vacancy (NV) centre in diamond is a promising candidate for quantum computing applications and magnetic sensing applications, because it is an atomic-scale defect with stable coherence time (*T*_2_) and reliable accessibility at room temperature. We demonstrated a method for improving the NV spin properties (the full width half maximum (FWHM) value of the magnetic resonance spectrum and *T*_2_) through a near-field (NF) etching method under ambient conditions. The NF etching method, based on a He-Cd ultraviolet laser (325 nm), which is longer than the absorption edge of the oxygen molecule, enabled selective removal of defects on the nanodiamond surface. We observed a decrease in the FWHM value close to 15% and an increase in *T*_2_ close to 25%. Since our technique can be easily reproduced, a wide range of NV centre applications could be improved, especially magnetic sensing applications. Our results are especially attractive, because they have been obtained under ambient conditions and only require a light source with wavelength slightly above the O_2_ absorption edge.

## Introduction

The nitrogen-vacancy (NV) centre has emerged as a promising candidate for nanoscale sensing applications in physics and biology^1–5^. Here, sensing is commonly realized by reading out the NV spin resonances (e.g., optically-detected magnetic resonance (ODMR)) and its shift under magnetic fields, with the magnetic sensitivity being dependent on the NV coherence properties^6^. However, sensing can also be realized by directly observing the change in the NV coherence time when close to magnetic molecules^7^. This spin-coherence-reliant approach has been well summarized under the term quantum sensing^8^. However, for either approach, the importance of *T*_2_ prevails.

Furthermore, to bring quantum computing closer to reality, it is critical to find a qubit candidate with long enough coherence times. The coherence time (*T*_2_) is the time for which the system stays in a superposition, and can thus be effectively used for the transmission of quantum information. One of the criteria—introduced by D. DiVincenzo for a system to perform quantum operations—is a coherence time that is much longer than the gate operation time^9^. Unfortunately our current qubits are relatively volatile, meaning that they lose their quantum information rather quickly. Hence, the current goal is to push coherence times to their maximum. Although it was possible to achieve coherence times well into the minute range, by using ionized donors combined with optical methods and dynamical decoupling^10^, it is still of common interest to find a more convenient qubit candidate, which can be used under ambient conditions. One of these candidates is the NV centre in diamond, which naturally exhibits excellent characteristics, such as long electron-spin coherence and relaxation times as well as unique optical and magnetic properties^11–16^. The NV centre can be initialized, driven, and read out^17–19^ by using external optical and microwave fields. Under room temperature, the *T*_2_ of the NV centre typically lies in the microsecond range^20^, with the benchmark currently being 1.8 ms achieved by using an ultrapure isotopically controlled single-crystal chemical vapour deposition (CVD) method^21^. From a material point of view, this can be done by purifying the diamond first by decreasing the amount of additional defects in the crystal. It can further be purified by isotopically engineering the carbon and making the effort to have only the nuclear-spin-free ^12^C isotopes in the crystal^22^. A previous research group has successfully improved the coherence properties of diamond bulk NV-centres via surface etching (wet oxidative chemistry combined with annealing at 465 °C^23^). However, so far there have been no reports on the effect of etching on the coherence properties of nanodiamond NV centres, although nanodiamonds are crucial for sensing applications^24^.

*T*_2_ is limited by fast fluctuations (that cannot be refocused by Hahn-echo), which can originate from flip-flops of spins at the surface of the diamond. Hence, we assume that *T*_2_ is directly proportional to the amount of noise on the surface of the diamond. It should be noted that a recent study by Knowles *et al*.^25^ suggested that the main contribution to the NV centre decoherence comes from nearby nitrogen impurities; however, in this paper, we would like to focus on improving the surface conditions, as this could also be useful for applications not based on the NV centres. Besides the known methods for reducing the impact of surface noise, such as dynamical decoupling^26^, previous studies indicate that ultraviolet (UV) lasers (325 nm) can induce ultra-fine surface etching (near-field (NF) etching) on various samples, including diamond, under ambient conditions^27^. We demonstrated that under sufficiently long-wavelength UV laser-light (325 nm) illumination, it was possible to reduce the dimensions of the nanodiamonds, alongside a decrease in the full width at half maximum (FWHM) value of the ODMR spectrum, and an increase in *T*_2_. These results indicate that the surface etching achieved with the NF etching method results in the removal of parasitic magnetic fields and optical noise from the nanodiamond surface.

## Results

### Structural change in nanodiamonds through a near-field etching approach

To improve the spin properties of nanodiamond NV, we performed NF etching on the NV nanodiamonds. Under far-field light irradiation, charges are generated in sub-wavelength objects (nano-scale surface protrusions), resulting in the generation of optical near-fields (ONF), which interact with matter within sub-wavelength distances (such as gas molecules). ONFs have non-uniform electric fields, which can excite dipole-forbidden states of molecules and trigger second harmonic generations^28^. Because the laser photon energy is lower than the O_2_ binding energy (5.12 eV), the presence of ONFs is required for the dissociation of O_2_ molecules^29–33^. In other words, the selectiveness of the photo-dissociation enables a controlled etching of the surface protrusions. Previous studies already showed that this technique is able to etch and improve various substrates, including diamond substrates^34^ as well as nanodiamonds^35^.

**Figure 1:**
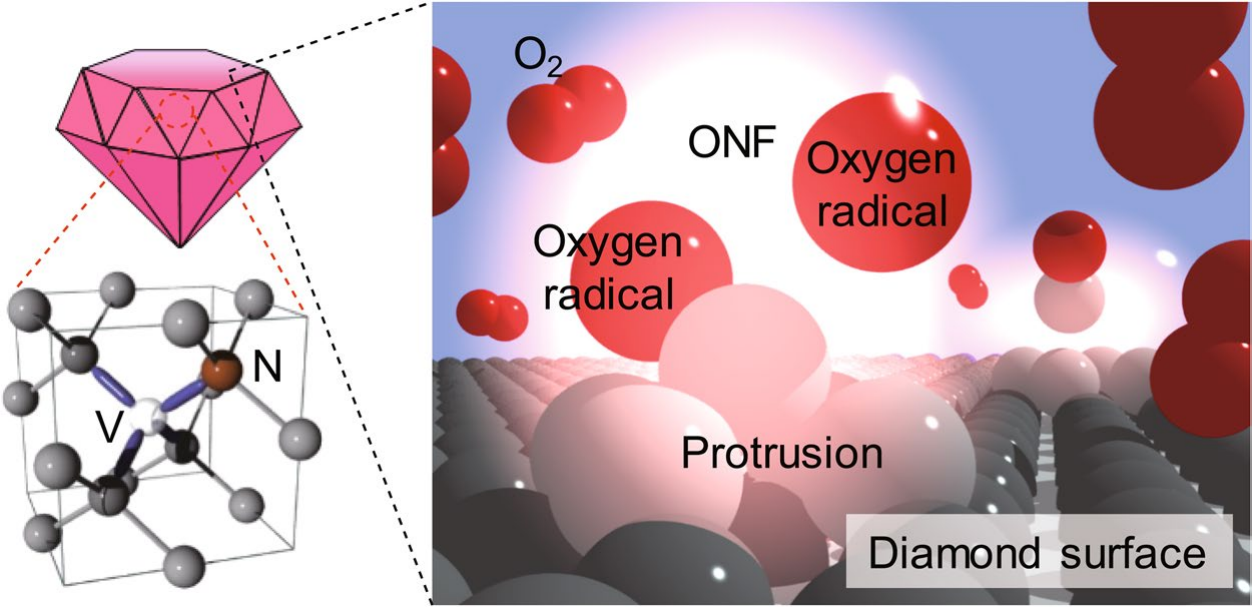
A schematic view of NF etching on the NV nanodiamonds. Optical near-fields (ONF) are generated by the He-Cd laser (3.81 eV) irradiation at the nanoscale protrusions on the nanodiamond surface. Since the photon energy is below the O_2_ dissociation energy (5.12 eV), O_2_ is only dissociated at the protrusions, thus allowing selective etching.

In the NF etching experiment (Fig. 1), the NV nanodiamonds with a diameter of approximately 200 nm^36^ were irradiated by a He-Cd laser with a wavelength of 325 nm (3.81 eV, lower than the dissociation energy of O_2_, 5.12 eV) with 8 mW under atmospheric conditions and ambient O_2_ molecules as the source for the etching effect. The nanodiamonds (see Methods) were dispersed on a Si substrate before being perpendicularly illuminated with a UV laser. While being illuminated with the UV laser, the nanodiamonds appeared to be etched on their surface and, over time, slowly reduced in size. Prior to evaluating how NF etching affects the spin properties of nanodiamond NV, we first evaluated the general effectiveness of NF etching on nanodiamonds, i.e., checking the degree of structural change.

**Figure 2:**
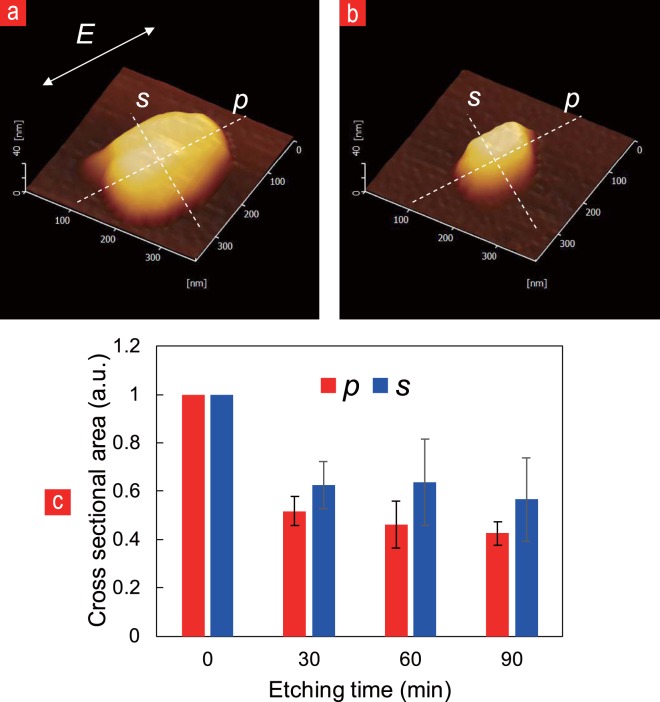
AFM images of the nanodiamonds, comparing their size before (**a**) and after 90 min (**b**) of He-Cd laser illumination. The comparison in size has been further examined through AFM in (**c**) by comparing the cross-sectional area of the nanodiamonds along the presumed parallel laser polarization axis (*p*-axis) and the presumed perpendicular laser polarization axis (*s*-axis), showing a general decrease in the cross-sectional area over illumination time.

Figure 2 shows the extent of the size reduction of the nanodiamond during He-Cd laser illumination. The two dashed-lines over the nanodiamonds represent the parallel-axis (*p*) and perpendicular-axis (*s*) to the He-Cd laser polarization (*E*). The atomic force microscope (AFM) images of the nanodiamond show a significant size reduction over a total timespan (90 min) of He-Cd laser illumination (Fig. 2a,b). In Fig. 2c we observe the average change of eight individual nanodiamonds for the cross-sectional area along the *p*-axis and *s*-axis over time (0, 30, 60, and 90 min), since previous research^37^ indicated the structural change to be caused mainly along the laser polarization-parallel-axis (*p*). In accordance with the previous study^38^, the initial 30 min apparently account for the highest etching rate, while after that, the NF etching effect appears to saturate (Fig. 2c). Within the first time interval (0–30 min) the area has been roughly halved (52% (*p*-axis) and 62% (*s*-axis)). Nevertheless, for continued illumination longer than 30 min, the extent of size reduction caused by NF etching appears more subtle, with the (30–60 min) and (60–90 min) intervals showing a size reduction along the *p*-axis to 46% and 42% and along the *s*-axis to 63% and 56%, respectively.

### Evaluation of the effect of NF etching on NV spin properties

**Figure 3:**
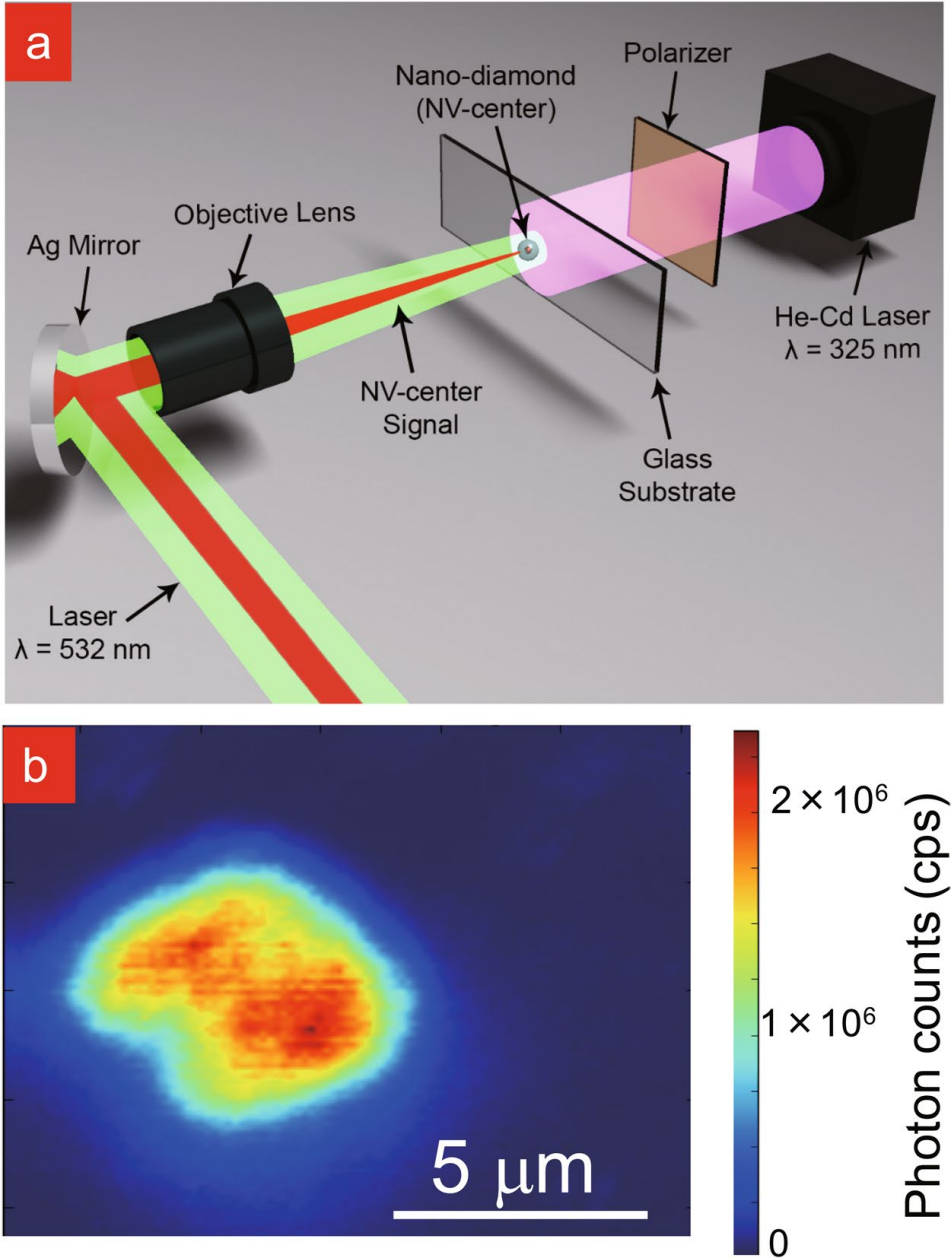
(**a**) Schematic view of the experimental setup. (**b**) Typical 2D fluorescence mapping of NV nanodiamonds (500 NV).

To evaluate the effect of NF etching on the NV nanodiamonds, we compared the ODMR- and Hahn-echo *T*_2_ signal, through the confocal setup, for an individual nanodiamond between the before and after NF etching cases (Fig. 3a). This sequence was repeated every 30 min, thus enabling us to observe slow changes in nanodiamond size and NV spin properties with etching time for the same nanodiamonds (Fig. 3b).

**Figure 4:**
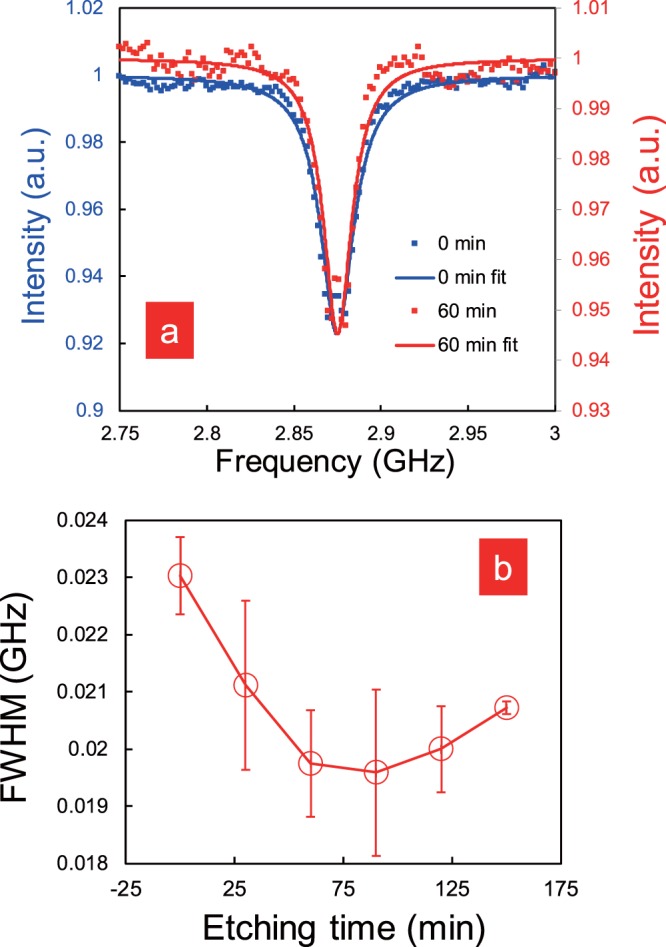
(**a**) ODMR spectra before (blue solid line (left-hand vertical axis); 0 min.) and after (red solid line (right-hand vertical axis); 60 min) NF etching. (**b**) FWHM value of the ODMR spectrum as a function of the NF etching time.

Figure 4a shows the time-averaged ODMR spectra for 500 s. Note that all ODMR spectra were obtained from the same nanodiamond containing 500 NV. As shown in Fig. 4a, we observed a decrease in the FWHM of the ODMR spectrum with increasing etching durations. By single Lorentzian fitting of the raw ODMR spectrum in Fig. 4a, we gained a more detailed perspective of the effective change in the FWHM values, as shown in Fig. 4b. The dots represent its average FWHM value. The error bar hereby represents the variance of the raw data points from their Lorentzian fitting in Fig. 4a. Figure 4b shows that the initial FWHM of approximately 23 MHz decreases gradually until it bottomed at around 19.6 MHz after an etching time of 90 min (~15% reduction compared to the initial value). However, longer NF etching times seem to have a somewhat opposite effect on the FWHM value, increasing it up to approximately 20.5 MHz for etching times close to 150 min. Although the FWHM was reduced, the contrast was reduced as well. The reduction of the contrast should be due to the reduction of the number of NV centres along the size reduction of the nanodiamonds. However, if we prepare a larger nanodiamond and take into account the NF etching depth, we could achieve a similar contrast as that before the etching. Therefore, we believe that a higher sensitivity with the reduction of FWHM can be achieved while keeping the ODMR contrast constant.

**Figure 5:**
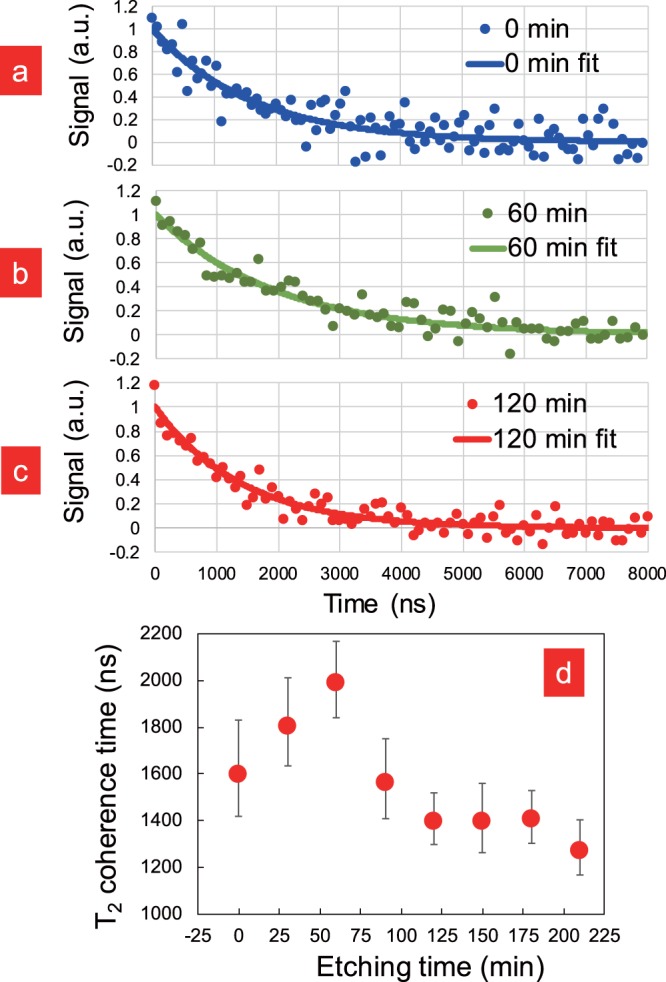
Normalized Hahn-echo signals (dots) before (**a**), after 60 min (**b**), and after 120 min (**c**) of NF etching, with exponential fitting curves (lines). (**d**) The measured Hahn-echo *T*_2_ of the single-NV nanodiamond as a function of the NF etching time.

Figures 5a–c show the typical Hahn-echo signal before and after NF etching. Each signal was accumulated for 1000 s. Note that all the Hahn-echo signals were obtained from the same nanodiamond containing single NV. Figure 5b appears to have a longer coherence time (*T*_2_) than Figs. 5a and 5c. From our original coherence data (splatters in Figs. 5a–c, we extracted *T*_2_ by fitting the data to an exponential function *f*(*t*) = exp[−*t*/*T*_2_]. To visualize the improvement in *T*_2_, we plotted *T*_2_ as a function of the NF etching time, as shown in Fig. 5d. The nanodiamonds used for the Hahn-echo measurement contained only a single NV centre on average and the error bar represents the variance of the raw data from their exponential fit in Figs 5a–c. Results in Fig. 5d show that *T*_2_ increases after being exposed to NF etching. The initial *T*_2_ (~1600 ns) improved with increasing NF etching times and peaked (~2000 ns) after approximately 60 min, corresponding to an increase of approximately 25%. After that *T*_2_ dropped significantly with longer NF etching durations until it hit bottom at approximately 1400 ns (~87.5% of its initial value) for etching times longer than 120 min.

## Discussion

Our results demonstrate that the FWHM value as well as the spin coherence time *T*_2_ can both be improved within an early timeframe of NF etching. The results from Figs 4b and 5d suggest that when applying NF etching, there is a specific exposure time, which will result in optimal values for both the FWHM value and spin coherence time *T*_2_.

The change in the FWHM value (Fig. 4b) can be explained as follows. The ODMR spectrum depends strongly on the presence of paramagnetic noise on the nanodiamond surface, especially when the NV centre exists close to the diamond surface^39^, though generally, the FWHM value cannot be reduced beyond a certain limit, which is set by the internal distortion of the nanodiamond.

The change in *T*_2_ (Fig. 5d) can be explained in a manner similar to the FWHM change. It should be derived from the change in the magnetic environment surrounding the NV centre. NF etching has the ability to remove surface impurities, such as structural defects and termination groups, which are known to influence the NV spin. Surface defects create a defect level in the band gap of diamond, which will be occupied by an electron whose spin will influence the NV centre^40^. Surface termination^41^ groups can influence the NV centre as well, as previous research has found that atoms attached to the surface of the NV centre in diamond can affect its spin coherence time^42^. Dangling bonds on the nanodiamond surface are another problem, since they are not able to covalently bond with another partner and are thus occupied by unpaired electrons with spins^43^. Oxygen molecules adsorbed onto the diamond surface can affect the NV-centre spin due to their paramagnetic nature^44^. Thus, the initial increase in *T*_2_ can be explained by the removal of surface defects and termination groups. On the other hand, the decrease in *T*_2_ could have been caused by an increase in the number of dangling bonds and oxygen molecules adsorbed onto the nanodiamond surface. Eliminating the termination group, as well as removing carbon atoms, leaves energetically unstable dangling bonds on the nanodiamond surface, attracting reactive compounds in the air, which further affect the NV spin. Although NF etching visibly changes the structure of the nanodiamonds, we can generally exclude the possibility that the single NV centre was removed (due to the measured NV signal over the whole etching distance, Fig. 5). The different peak times in Figs 4b and 5d can be explained by the usage of different nanodiamond types. The NV-dense nanodiamond was more suitable for ODMR measurements than the single NV nanodiamond (which, on the other hand, is more interesting for applications of *T*_2_).

In conclusion, we demonstrated that NV nanodiamond exposure to NF etching under ambient conditions can reduce the FWHM value by up to 15% and increase *T*_2_ by up to 25%. Magnetic sensing applications could be improved, since they depend on the ODMR quality as well as *T*_2_. Most importantly, due to the simplicity of the experiment, NF etching has the potential to improve numerous applications based on spin coherence.

For future experiments, by examining the exact role of the far-field light parameters, such as wavelength, power density, and duration, we could get a clearer understanding of how frequent localized near-fields are formed, and ultimately use them for precise structure and surface conditioning.

## Methods

### Nanodiamond sample preparation

The nanodiamond solution was purchased from Academia Sinica (rFND-OH, 0.1% w/v) and FND Biotech, Inc. (cFND, 1 mg/mL, -COOH). The rFND (typically 50 nm, containing single NV) were used for the AFM and *T*_2_ measurements, while the cFND (typically 200 nm, containing approximately 500 NV, obtained through milling (8000 M, SPEX) of microdiamond powder^45^) were used for the FWHM measurements. Both nanodiamond types were initially covered by hydroxyl groups. The nanodiamond solutions were dropped on a 2 × 2 cm silicon substrate, which was previously cleaned using a 1:1 mixture of sulphuric acid and hydrogen peroxide at 190 °C for approximately 15 min. After dropping the nanodiamond solution onto the Si substrate, we placed the sample on a hot plate at 160 °C causing the water to evaporate.

### Near-field etching of nanodiamond

The nanodiamonds were etched by the NF etching method, where induced ONFs at sub-wavelength protrusions are assumed to dissociate O_2_ molecules, resulting in oxygen radicals (Fig. 1). For this purpose, they were vacuum-fixed onto the AFM stage, to ensure minimal misalignment of the nanodiamonds within the AFM measurement windows between each etching interval. In case of misalignments, an inbuilt function allows the AFM stage control to refocus on the specific nanodiamonds. For the NF etching, a continuous-wave (CW) He-Cd laser (325 nm; 3.81 eV; excitation power density: 0.8 W/cm^2^) was chosen because its energy was below the bonding energy of O_2_ (5.12 eV), thus avoiding conventional adiabatic etching. The emission line of the He-Cd laser for other wavelengths (outside 325 nm) is with a factor of around 10^−4^, which is negligibly low and can therefore be ignored. The nanodiamond-hosting silicon plane was perpendicularly illuminated by the He-Cd laser for 30-min intervals between each measurement (structural change, *T*_2_, and ODMR measurements).

### AFM evaluation

The sample images were obtained by using an AFM (Hitachi Hitech Science Corp.). All nanodiamond images were obtained within an area of 3 μm with a resolution of 256 × 256 pixels. A special “sample intelligent scan” mode was used during the measurement to reduce unwanted noise and also to improve image quality by using tilt compensation features.
